# Supplementary material for: Active label cleaning for improved dataset quality under resource constraints
Source: Nat Commun. 2022 Mar 4;13:1161. doi: 10.1038/s41467-022-28818-3 (PMC8897392; doi:10.1038/s41467-022-28818-3)
Supplement: Supplementary file 1 — Supplementary Information [file 41467_2022_28818_MOESM1_ESM.pdf]

Active label cleaning for improved dataset quality under resource constraints

Supplementary information

Mélanie Bernhardt[1, †], Daniel C. Castro[1, †], Ryutaro Tanno[1], Anton Schwaighofer[1], Kerem C. Tezcan[1], Miguel Monteiro[1], Shruthi Bannur[1], Matthew P. Lungren[2], Aditya Nori[1], Ben Glocker[1], Javier Alvarez-Valle[1], and Ozan Oktay[1, *]

[1] *Health Intelligence, Microsoft Research Cambridge, Cambridge, CB1 2FB, UK*
[2] *Department of Radiology, Stanford University, Palo Alto, CA 94304, USA*

[†] *These authors contributed equally to this work.*
[*] *Corresponding author: ozan.oktay@microsoft.com*

Supplementary Note 1: Additional experiments

Classification performance of noise-robust models. We test the robustness of the proposed approaches to the corrupted labels in the training set and measure how well they generalise to an unseen set with clean labels. To this end, all networks are trained on a dataset $\mathcal{D}_{\text{train}} = \{(\mathbf{x}_i, y_i)\}_{i=1}^{N}$ containing both mislabelled and correctly labelled data points. To assess the robustness to the noisy labels, we separate $\mathcal{D}_{\text{train}}$ into disjoint subsets of correctly labelled ($\mathcal{D}_{\text{corr}}$) and mislabelled cases ($\mathcal{D}_{\text{misl}}$). Similarly, the robustness is measured on a hold-out evaluation set ($\mathcal{D}_{\text{eval}}$) with clean labels.

We analyse the classification metrics for these three groups separately throughout training for the different proposed methods. The results are reported in supplementary Table 1 in terms of multi-class classification accuracy (CIFAR10H) and ROC-AUC values for binary classification (NoisyCXR). In this analysis setup, robustness means that the network should learn to ignore the incorrect labels of the mislabelled cases and yield an accuracy close to chance for these. On the other hand, this should not come at a price of reduced performance for the correctly labelled cases and the network should yield a high accuracy for these. Hence the ideal case would be a large gap between the accuracy of these two groups ($\mathcal{D}_{\text{corr}}$, $\mathcal{D}_{\text{misl}}$) throughout training and the gap is a good proxy measure of sample selectors' performance in the relabelling procedure.

Supp. Table 1 The models used in the simulation experiments are also evaluated in terms of their classification performance on images with clean and noisy labels.

Dataset	Model	Acc on $\mathcal{D}_{\text{eval}}$	Acc on $\mathcal{D}_{\text{corr}}$	Acc on $\mathcal{D}_{\text{misl}}$
CIFAR10H	Vanilla	77.22 (0.29)	92.38 (0.20)	45.50 (2.78)
	Co-teaching	79.19 (0.22)	88.38 (0.26)	26.79 (1.64)
	SSL + Linear head	80.56 (0.11)	85.37 (0.20)	12.62 (0.44)
		AUC on $\mathcal{D}_{\text{eval}}$	AUC on $\mathcal{D}_{\text{corr}}$	AUC on $\mathcal{D}_{\text{misl}}$
NoisyCXR	Vanilla	83.52 (0.85)	89.87 (0.42)	32.71 (1.86)
	Co-teaching	84.91 (0.31)	90.17 (0.26)	28.10 (0.29)
	SSL + Co-teaching	87.45 (0.02)	91.84 (0.16)	25.92 (0.19)

The networks are trained on a dataset containing partly mislabelled images: CIFAR10H: $|\mathcal{D}_{\text{train}}| = 10k$, $\eta = 15\%$; NoisyCXR: $|\mathcal{D}_{\text{train}}| = 13.3k$, $\eta = 12.7\%$, where η is the noise rate. Their performance is evaluated on an evaluation set $\mathcal{D}_{\text{eval}}$ with clean labels, and training set with clean $\mathcal{D}_{\text{corr}}$ and noisy labels $\mathcal{D}_{\text{misl}}$ ($\mathcal{D}_{\text{misl}} \cap \mathcal{D}_{\text{corr}} = \emptyset$). The size of the evaluation sets is $|\mathcal{D}_{\text{eval}}| = 50k$ for CIFAR10H and $|\mathcal{D}_{\text{eval}}| = 13.3k$ for NoisyCXR. The results are aggregated over three separate runs with different seeds (std. deviation in parentheses) and reported in terms of classification accuracy (Acc) and ROC-AUC (AUC).

Simulation experiments with larger noise rates.

CIFAR10H Label cleaning simulations are repeated for larger noise rates to understand the sensitivity of selection algorithms to noise rate. This way we also explore the limits at which data-driven label cleaning algorithms may become unreliable. For this purpose, we use the same set of CIFAR10H images ($|\mathcal{D}| = 10k$) but with a different initial set of noisy labels sampled from the label distribution using a larger temperature parameter ($\tau > 2$). In this way, we are able to control the initial noise rate up to 34%. For noise values beyond this rate, a second parameter is introduced as an additive bias term applied to the confusion matrix, which can serve as a uniform symmetric or class-dependent noise model. At the end, we construct three different sets of initial labels with the following average noise rates and noise models: (I) 30% noise (CIFAR10H distribution), (II) 50% noise (CIFAR10H + symmetric), and (III) 50% (CIFAR10H + class-dependent).

For larger noise rates, the cost-effectiveness of sample selection algorithms are experimentally observed to be preserved as shown in supplementary Figs. 1a and 1b. It is important to note that in 30% noise case the breakdown of clear and difficult noisy cases are 23.24% and 6.66% respectively. On the other hand, when we switch to class-dependent noise model with more than 50% noise rate, the sample ranking quality begins to degrade as diagonal dominance is no longer preserved for some certain classes (e.g. dog, cat, deer, horse, and automobile). We expect the algorithms to degrade towards random sampling as we introduce further bias and noise into the initial set of labels.

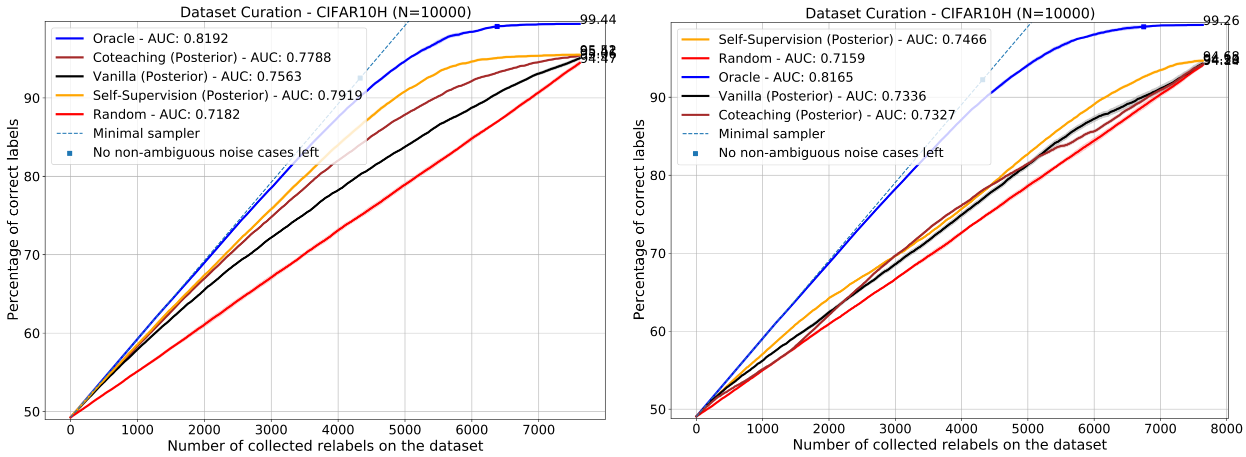

a CIFAR10H – Noise rate 50% Symmetric Noise

b CIFAR10H – Noise rate 50% Asymmetric Noise

Supp. Fig. 1 Label cleaning performance for noise rate of 50%, using symmetric noise and a more realistic, asymmetric noise model. Shaded areas represent +/- standard deviation over 5 random seeds for relabelling.

NoisyCXR In this section, we investigate the impact of higher noise rates on label cleaning simulations for NoisyCXR. To construct datasets with more noise, we followed the same procedure as described in Methods but instead of sampling 10% of noise within the NIH "Consolidation/Infiltration" category we sampled 25% noise within this category. Including both the NIH noise and the sampled noise, the dataset contained in total 20% noise. On this dataset, note that 43% of positive initial (noisy) labels are incorrectly labelled, making it inherently challenging to learn an accurate classifier on this noisy training dataset. With this higher noise rate, we observe that the sample selection quality of *co-teaching* degrade towards the *vanilla* model. On the other hand, we still observe that combining co-teaching with self-supervised pre-training improves the quality of the label cleaning procedure, as the model is able to separate noisy from clean cases meaningfully earlier in training, hence improving co-teaching.

Further analysis on the scoring function. To better understand the impact of each term in the scoring function Φ, we conducted simulation experiments by dropping the self-entropy term that captures the sample ambiguity. We hypothesise that this term encourages selectors to give a higher priority to clear label noise cases over the samples with higher labelling difficulty. To experimentally verify this, we first split the noisy samples into two disjoint subsets: clear noisy and difficult noisy. This is done by thresholding the normalised entropy of each sample's true label distribution at 0.3. Then we tracked the size of both noisy sets throughout the active label cleaning procedure to understand the sample ranking pattern. The experiments are conducted on the CIFAR10H dataset with 30% and 50% noise rates. The corresponding results are shown in supplementary Fig. 4. We observe that by including the self-entropy term, the *SSL* selector first prioritises the clear label noise cases as highlighted by the difference between green and orange curves in the plot (middle and right), which also yields a slight performance improvement in terms of mislabel correction rate. Moreover, we observe that the best performing sample selectors favour correcting first the clear label noise cases as it can be seen by the difference between the *SSL* and *Vanilla* approaches.

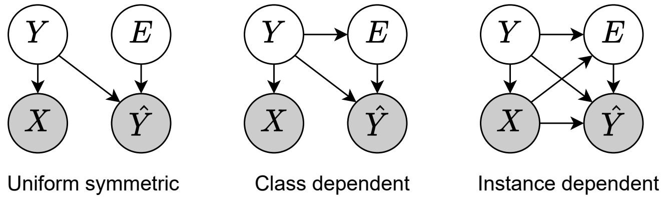

Supp. Fig. 2 Different label noise models used in robust learning. The statistical dependence between input image (X), true label (Y), observed label ($\hat{Y}$), and error occurrence (E) is shown with arrows. Adapted from Frénay et al.[1].

Model ensembles yield better aleatoric uncertainty estimates. The ambiguity term in the scoring function (Φ) can be better estimated when the posterior entropy is marginalised over a weight distribution as in the case of BALD[3]. To verify this hypothesis, an ensemble of SSL models are formed by training with 5 different seeds of weight initialisation whilst using the same set of initial labels. The simulations are repeated 5 times with different label sampling patterns. Supp. Table 2 provides the AUC values for these experiments, where we see that the ambiguity term (Ensemble-Ent) improves the results further when ensembles are used and averaged posteriors (Ensemble) do not yield drastic improvement. supplementary Fig. 6 shows this comparison for different resource constraint (B) values.

Impact of model update frequency: An ablation study is conducted to understand the influence of updating model's beliefs during the relabelling process (see supplementary Table 3). In that regard, experiments are repeated for different b values which determines the frequency of model updates using the collected labels. With these updates, an improved performance is observed on all model based approaches, which converge towards

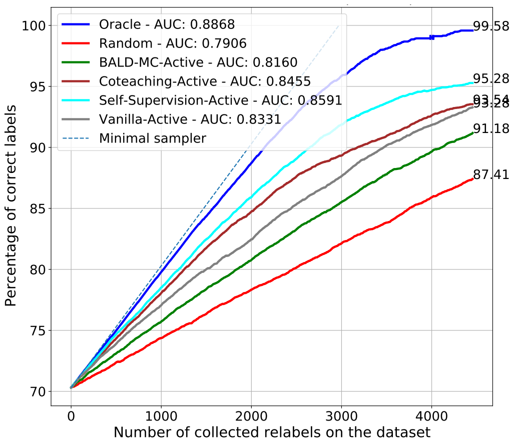

a CIFAR10H with η = 30% initial noise rate

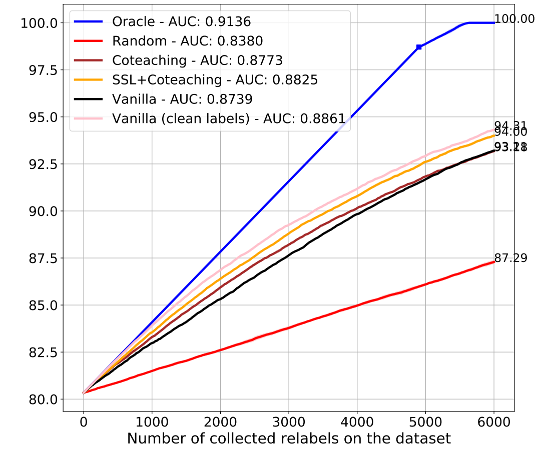

b NoisyCXR with η = 19.7% initial noise rate

Supp. Fig. 3 Correctness of labels (y-axis) with respect to re-labelling budget (x-axis) for higher noise rate for CIFAR10H and NoisyCXR. Fig (a) shows that standard active learning based sample scoring functions, such as BALD[2] (in green), do not necessarily prioritise noisy labels in the relabelling procedure. Shaded areas represent +/- standard deviation over 5 random seeds for relabelling.

Supp. Table 2 Impact of model ensembles on label cleaning performance and uncertainty estimation

	(AUC) Perc. of correct labels vs number of relabels			
Model	Single	Single-Ent	Ensemble	Ensemble-Ent
SSL	.818	.819	.820	.823

The simulations are run over 5 different seeds of annotation sampling procedure. To form an ensemble, 5 different models are trained with different weight initialisations whilst keeping the initial labels the same (CIFAR10H, η = 30%, B = 3k).

the upper-bound set by a model trained with all clean labels.

Instance-dependent noise model. Uniform and class-dependent noise models, illustrated in supplementary Fig. 2, do not have a direct statistical dependency on the image content. This may lead researchers to design and experiment with artificial noise models that may not always hold true in practical scenarios. For instance, it would be more difficult to label low-resolution images of brown horses in comparison to white ones, since class confusion probability between horse and deer is likely to be higher.

To model such dependencies, recent work has proposed the use of instance-dependent noise (IDN) models[4,5]. In our study, we re-implemented the IDN model proposed in[5] (Algorithm 2), where instance-specific flip rates $\mathbf{p}_i \in \mathbb{R}^C$ are computed for each instance i using a set of weights $\{\mathbf{W}_1, \mathbf{W}_2, \ldots, \mathbf{W}_C\}$ as $\mathbf{p}_i = \mathbf{W}_{y_i}\,\mathrm{vec}(\mathbf{x}_i)$. The weights $\mathbf{W}_c \in \mathbb{R}^{C\times L}$ are drawn from a normal distribution, $\mathrm{vec}(\mathbf{W}_c) \sim \mathcal{N}(0, \mathbf{I})$. The individual class flip probabilities are later softmax-normalised across all classes and scaled with the desired noise rate to generate instance-specific class confusion matrices. In contrast to[5], here we utilise image embeddings generated with a pretrained image model (ResNet-50, ImageNet) instead of raw pixel values $\mathbf{x}_i$. This is mainly intended to associate the generated class transition models with higher-level image semantics.

Supp. Table 3 Ablation study on the frequency of model updates (b) in active relabelling procedure.

		% of Correct Labels (std) and AUC			
	Model	b = 500	b = 1000	b = 2000	b = ∞
NoisyCXR	Oracle	-	-	-	100.0 (0), .946
	Vanilla	94.81 (.08) .916	94.82 (.04) .915	94.75 (.05) .914	94.34 (.03) .913
	SSL	95.12 (.04) .919	95.04 (.04) .919	95.02 (.04) .918	95.03 (.02) .918
CIFAR10H	Oracle	-	-	-	99.38 (.19), .887
	Vanilla	93.23 (.11) .836	93.14 (.25) .836	92.72 (.36) .834	91.27 (.34) .829
	SSL	95.21 (.20) .861	95.12 (.19) .860	94.97 (.19) .858	94.94 (.17) .858

Model update frequency impact on the number of corrected labels is assessed on CIFAR10H (30% initial noise, B = 4.5k) and NoisyCXR (12.7% initial noise, B = 4k) for *vanilla* and *SSL-Linear*. The simulations are run over 5 different seeds of model fine-tuning and label sampling.

Supplementary Note 2: Implementation Details

CIFAR10H.

Supervised models: For all experiments, the classifier and selector are trained with the same model configuration but they differed in terms of the labels used to train them. For all experiments, we trained a ResNet-50 model with a bottleneck unit containing convolution layers with batch-normalisation. The models are trained for 160 epochs with the SGD optimiser and Nesterov momentum (0.9) using a base learning rate of 0.1 and weight decay of 10^{-4}. The learning rate is reduced by a factor of 0.1 at epochs 80 and 120. Each gradient update is computed over a mini-batch of 256 input images by computing cross-entropy with respect to smoothed target labels ($\epsilon = 0.1$). Images are augmented prior to model forward pass by applying standard natural image transformations (e.g. colour jitter, affine transformation, and horizontal flips).

For the training of co-teaching models[6], the drop-rate is set to

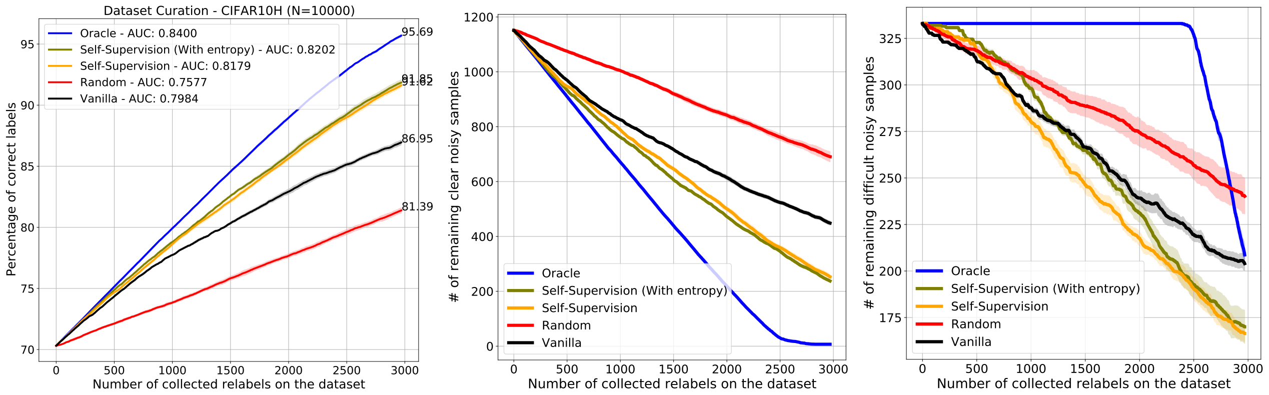

a CIFAR10H – Noise Rate 30%

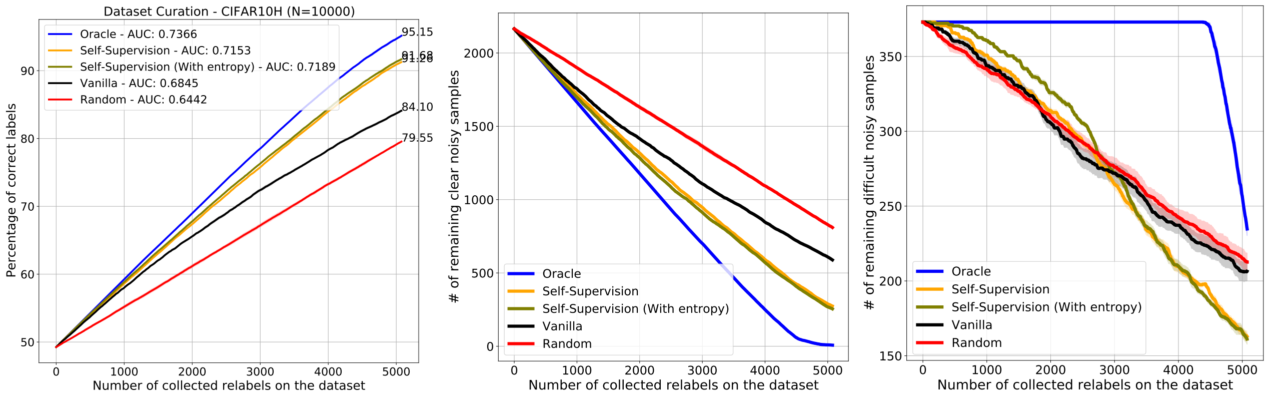

b CIFAR10H – Noise Rate 50%

Supp. Fig. 4 Results of noisy label cleaning on CIFAR10H dataset with different noise rates. The figures on the left illustrate the percentage of correct labels with respect to the number of relabels collected in the simulation. Here we see that by including the self-entropy term in *SSL* selector (green), the final outcome can be slightly improved (91.85 vs 91.02). Similarly, the number of remaining clear and difficult label noisy cases are shown in the middle and right, respectively. Best performing methods prioritise clear label noise cases over the ones with more labelling difficulty. Lastly, we see that this behaviour is further emphasised by including the ambiguity term in the sample scoring function (green vs orange curves). Shaded areas represent +/- standard deviation over 5 random seeds for relabelling.

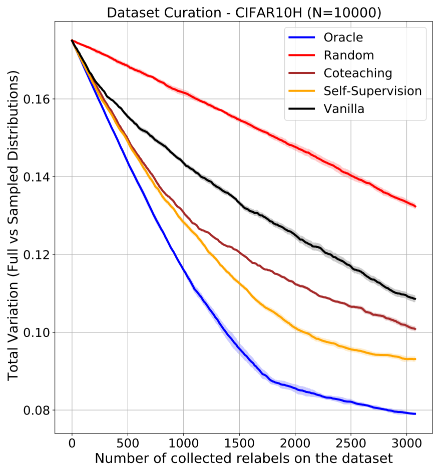

Supp. Fig. 5 Total variation (y-axis) between true label distribution and distribution of sampled labels in function of relabelling budget (x-axis). A similar performance ranking is observed as in the experiments measuring the accuracy of majority labels (CIFAR10H, η = 30%). Shaded areas represent +/- standard deviation over 5 random seeds for relabelling.

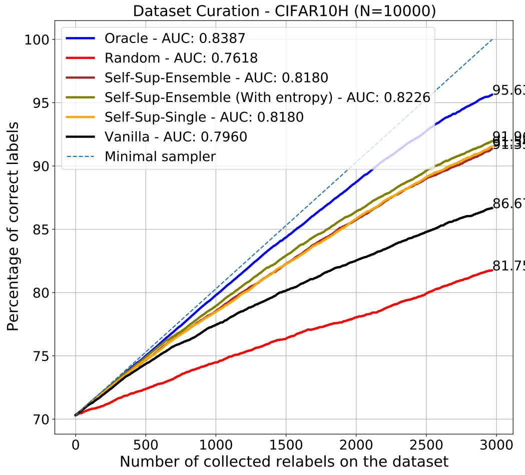

Supp. Fig. 6 Simulation experiment benchmarking sample selectors that are based on single SSL model and ensemble of them. Uncertainty estimates become more reliable (green curve) when the entropy term is marginalised over model weights (CIFAR10H dataset, η = 30%). Shaded areas represent +/- standard deviation over 5 random seeds for relabelling.

match the expected noise rate in the datasets, which can be further tuned on a separate validation set for potentially improved performance. In the initial warm-up phase (up to epoch 10), all samples are used in SGD updates, including the ones with large loss value.

SSL training: For the training of BYOL[7] self-supervision models, CIFAR10H dataset ($10k$) is used as a training set. For each true image pair, the standard image augmentations are applied, which are outlined in detail in the SimCLR study[8]. The encoder models are trained with Adam[9] optimiser for 2500 epochs with the base learning rate of 10^{-3} and batch size of 512. As a learning rate scheduler, a cosine decay function is utilised. More importantly, in order not to bias the model comparison, the same ResNet-50 architecture is used as the base encoder for the BYOL models.

To build an image classifier on top of *SSL* embeddings, a single linear layer is trained for 120 epochs using both weight decay (10^{-3}) and tanh logit regularisation terms ($\alpha = 20$, defined in Methods) in order to avoid over-fitting on the noisy labels. Similarly to the NoisyCXR experiments, co-teaching could potentially be utilised together with *SSL* weights without freezing the encoder parameters, which is omitted in CIFAR10H experiments as the linear head experimentally proved to be sufficient in identifying noisy labels.

NoisyCXR.

Supervised models: As for CIFAR10H, the classifier and selector are trained with the same model configuration but they differed in terms of the labels used to train them. For all experiments, a ResNet-50 model is trained with the Adam optimiser[9] with cross-entropy loss, using a learning rate of 10^{-5} for models trained from scratch (resp. 10^{-6} for fine-tuning), weight decay of 10^{-4} and batch size 32. For all models, we use 50% of the data for training and 50% for validation. At training time, the under-represented class ("Pneumonia-like opacity") is oversampled to handle the class-imbalance according to the class distribution observed in the training set. In terms of preprocessing, images are first augmented in native resolution (if applicable), then resized to 256×256, and finally they are centre-cropped to 224×224. At training time, the following augmentations are used: random horizontal flipping ($p = 0.5$), random rotation ($[-30°, 30°]$), random shear transform ($[-15, 15]$), random contrast, random brightness, and random crop ($[80\%, 100\%]$). Models trained from random initialisation are trained for 150 epochs; models initialised from a pre-trained checkpoint are fine-tuned for 100 epochs. For "SSL-Linear", we built a linear image classifier on top of the frozen SSL encoder, trained for 100 epochs with a starting learning rate of 10^{-4} decreased to 10^{-5} after 10 epochs; we used tanh logits regularization ($\alpha = 10$) and reduced the set of augmentations to horizontal flips.

For the training of co-teaching models[6], the drop-rate is set to match the expected noise rate in the datasets. The warm-up phase was set to 20 epochs during which no samples are dropped (resp. 10 epochs when initialised from SSL checkpoint).

Supp. Table 4 The impact of image augmentations on the quality of learnt BYOL embeddings, when applied to medical images.

Augmentations	ROC-AUC
All w/out cropping	0.853
All w/out brightness and contrast	0.866
All w/out rotation and shear	0.868
All augmentations (baseline)	0.871

The embedding quality is assessed in terms of linear separation of classes by training a linear-head on top of BYOL encoder output. At each experiment, one data augmentation is omitted to measure its impact, and classification results are obtained on the validation set using ROC-AUC.

SSL training: The self-supervised models have been trained with BYOL[7] using a ResNet-50 encoder. The final model is trained using the NIH dataset[10] (80% training, 20% validation), using an effective batch size of 4800 (batches of 600 pairs of images of size 224×224 on 8 GPUs) for 1000 epochs. The momentum parameter τ used in BYOL teacher encoder was set to 0.99. As augmentations, we use random horizontal flipping (with probability $p = 0.5$), random rotation and shear (drawn from $[-180°, 180°]$ and $[-40, 40]$), random crop ($[40\%, 100\%]$), CutOut (masking out rectangles of varying size, size covering 15–40% of the image), elastic transforms[11] ($a = 34$, $s = 4$, applied with probability 0.5), random contrast and brightness changes, and adding Gaussian noise ($\sigma = 0.01$, applied with probability 0.5).

We ran experiments on the NoisyCXR dataset to determine the impact of each of those augmentations on the quality of the resulting embedding. To this end, a linear classifier is learnt on top of image embeddings during BYOL training and its performance is monitored with ROC-AUC metric on the validation set. By disabling one augmentation at a time and comparing to the baseline with all augmentations, we see that image cropping contributes most to the results, followed by contrast and brightness augmentations, then rotation and shear, results are provided in supplementary Table 4. Using the full set of augmentations often meant that performance kept increasing when training for longer, whereas using fewer augmentations led to performance plateauing earlier in training. Histogram normalisation had a noticeable impact on the result. Using the native images without histogram normalisation led to an AUC roughly 0.6% higher than with histogram normalisation.

Supplementary References

1. Frénay, B. & Verleysen, M. Classification in the presence of label noise: a survey. *IEEE Transactions on Neural Networks and Learning Systems* **25**, 845–869 (2014).
2. Gal, Y., Islam, R. & Ghahramani, Z. Deep Bayesian active learning with image data. In *International Conference on Machine Learning*, 1183–1192 (PMLR, 2017).
3. Houlsby, N., Huszár, F., Ghahramani, Z. & Lengyel, M. Bayesian active learning for classification and preference learning (2011). URL https://arxiv.org/abs/1112.5745.
4. Chen, P., Ye, J., Chen, G., Zhao, J. & Heng, P.-A. Beyond class-conditional assumption: A primary attempt to combat instance-dependent label noise. In *Proceedings of the AAAI Conference on Artificial Intelligence*, vol. 35.

5. Xia, X. *et al.* Part-dependent label noise: Towards instance-dependent label noise. In *Advances in Neural Information Processing Systems*, vol. 33 (2020).
6. Han, B. *et al.* Co-teaching: Robust training of deep neural networks with extremely noisy labels. In *Advances in Neural Information Processing Systems*, 8527–8537 (2018).
7. Grill, J.-B. *et al.* Bootstrap your own latent - a new approach to self-supervised learning. In *Advances in Neural Information Processing Systems*, vol. 33, 21271–21284 (2020).
8. Chen, T., Kornblith, S., Norouzi, M. & Hinton, G. A simple framework for contrastive learning of visual representations. In *Proceedings of the 37th International Conference on Machine Learning*, 1597–1607 (PMLR, 2020).
9. Kingma, D. P. & Ba, J. Adam: A method for stochastic optimization. In *International Conference on Learning Representations* (2015). arXiv:1412.6980.
10. Wang, X. *et al.* ChestX-Ray8: Hospital-scale chest X-ray database and benchmarks on weakly-supervised classification and localization of common thorax diseases. In *2017 IEEE Conference on Computer Vision and Pattern Recognition (CVPR)*, 3462–3471 (2017).
11. Simard, P. Y., Steinkraus, D. & Platt, J. C. Best practices for convolutional neural networks applied to visual document analysis. In *Proceedings of the Seventh International Conference on Document Analysis and Recognition (ICDAR 2003)*, 958–963 (2003).
