# Supplementary material for: Active label cleaning for improved dataset quality under resource constraints
Source: Nat Commun. 2022 Mar 4;13:1161. doi: 10.1038/s41467-022-28818-3 (PMC8897392; doi:10.1038/s41467-022-28818-3)
Supplement: Supplementary file 3 — Reporting Summary [file 41467_2022_28818_MOESM3_ESM.pdf]

nature portfolio

Corresponding author(s): Ozan Oktay

Last updated by author(s): Feb 8, 2022

Reporting Summary

Nature Portfolio wishes to improve the reproducibility of the work that we publish. This form provides structure for consistency and transparency in reporting. For further information on Nature Portfolio policies, see our Editorial Policies and the Editorial Policy Checklist.

Statistics

For all statistical analyses, confirm that the following items are present in the figure legend, table legend, main text, or Methods section.

n/a	Confirmed	
☐	☒	The exact sample size (n) for each experimental group/condition, given as a discrete number and unit of measurement
☐	☒	A statement on whether measurements were taken from distinct samples or whether the same sample was measured repeatedly
☒	☐	The statistical test(s) used AND whether they are one- or two-sided<br>*Only common tests should be described solely by name; describe more complex techniques in the Methods section.*
☐	☒	A description of all covariates tested
☐	☒	A description of any assumptions or corrections, such as tests of normality and adjustment for multiple comparisons
☐	☒	A full description of the statistical parameters including central tendency (e.g. means) or other basic estimates (e.g. regression coefficient) AND variation (e.g. standard deviation) or associated estimates of uncertainty (e.g. confidence intervals)
☒	☐	For null hypothesis testing, the test statistic (e.g. F, t, r) with confidence intervals, effect sizes, degrees of freedom and P value noted<br>*Give P values as exact values whenever suitable.*
☐	☒	For Bayesian analysis, information on the choice of priors and Markov chain Monte Carlo settings
☐	☒	For hierarchical and complex designs, identification of the appropriate level for tests and full reporting of outcomes
☒	☐	Estimates of effect sizes (e.g. Cohen's d, Pearson's r), indicating how they were calculated

Our web collection on statistics for biologists contains articles on many of the points above.

Software and code

Policy information about availability of computer code

Data collection	Data was publicly available. We have not collected any data, all references to the datasets used in this study can be found in the Data Availability section.
Data analysis	The source code, which is used to analyze the datasets is available at https://github.com/microsoft/InnerEye-DeepLearning/tree/main/InnerEye-DataQuality. The repository contains the corresponding source files and related documentation. Out code contains all necessary information to replicate our experiments, including experiment configurations and code environment requirements.

For manuscripts utilizing custom algorithms or software that are central to the research but not yet described in published literature, software must be made available to editors and reviewers. We strongly encourage code deposition in a community repository (e.g. GitHub). See the Nature Portfolio guidelines for submitting code & software for further information.

Data

Policy information about availability of data

All manuscripts must include a data availability statement. This statement should provide the following information, where applicable:

- Accession codes, unique identifiers, or web links for publicly available datasets
- A description of any restrictions on data availability
- For clinical datasets or third party data, please ensure that the statement adheres to our policy

The CIFAR10H[5] dataset is available at "https://github.com/jcpeterson/cifar-10h"

The raw data for NoisyCXR can be downloaded from the RSNA Pneumonia Detection Challenge Kaggle page at "https://www.kaggle.com/c/rsna-pneumonia-detection-challenge/data"

The detailed annotations per annotator for this challenge can be downloaded from "https://storage.googleapis.com/kaggle-forum-message-attachments/844871/15572/RSNA_pneumonia_all_probs.csv", and the original labels from the NIH ChestXray dataset can be found under "https://s3.amazonaws.com/east1.public.rsna.org/AI/2018/pneumonia-challenge-dataset-mappings_2018.json"

The labels used in our experiments are processed in the linked code repository "https://github.com/microsoft/InnerEye-DeepLearning/tree/main/InnerEye-DataQuality"

The URLs were accessed on Feb 2nd, 2022

Field-specific reporting

Please select the one below that is the best fit for your research. If you are not sure, read the appropriate sections before making your selection.

- [x] Life sciences
- [ ] Behavioural & social sciences
- [ ] Ecological, evolutionary & environmental sciences

For a reference copy of the document with all sections, see nature.com/documents/nr-reporting-summary-flat.pdf

Life sciences study design

All studies must disclose on these points even when the disclosure is negative.

Sample size	The total of 26,684 frontal chest X-Ray scans from each individual patient (RSNA dataset) were eligible for inclusion in the work. The total sample size for training and validation sets was informed by the existing literature (Wang et al. CVPR'17, Majkowska et al. Radiology'20). In more detail, the dataset used in this study corresponds to the intersection of NIH-CXR8 and RSNA-CXR datasets, where multiple-labels are available for each data point. Having an access to multiple expert annotations is a requirement to carry out realistic annotation experiments as in the presented study.
Data exclusions	No clinical data were excluded from the analyses.
Replication	The experiments on both datasets were repeated for different (I) label noise rates, (II) initial label noise assignments. For each experiment we repeated the relabelling simulations with 5 independent CPU seeds. The results obtained where consistent across repetitions, for all seeds. Additionally, CIFAR10H experiments were repeated on different subsets of the CIFAR10 dataset to ensure that similar conclusions can be drawn from the simulations. To ensure reproducibility, we provide all necessary experiments configuration files in our code repository.
Randomization	Samples meeting the inclusion criteria were randomly allocated to training or validation sets. A separate group of patients were randomly selected before creation of the training and validation datasets as an independent test set which was kept separate during model development.
Blinding	Radiologists in the collection of data labels (clinical diagnosis) were blinded to the initial ground truth labels released by NIH (Wang et al. CVPR'17), and they were not involved in dataset collection. Please check the following web-link for further details: https://www.kaggle.com/c/rsna-pneumonia-detection-challenge/discussion/64723

Reporting for specific materials, systems and methods

We require information from authors about some types of materials, experimental systems and methods used in many studies. Here, indicate whether each material, system or method listed is relevant to your study. If you are not sure if a list item applies to your research, read the appropriate section before selecting a response.

Materials & experimental systems

n/a	Involved in the study	
x		Antibodies
x		Eukaryotic cell lines
x		Palaeontology and archaeology
x		Animals and other organisms
	x	Human research participants
x		Clinical data
x		Dual use research of concern

Methods

n/a	Involved in the study	
x		ChIP-seq
x		Flow cytometry
x		MRI-based neuroimaging

Human research participants

Policy information about studies involving human research participants

Population characteristics	For detailed information on the population characteristics, please check the web-links provided below:<br>https://nihcc.app.box.com/v/ChestXray-NIHCC/file/219760887468<br>https://www.rsna.org/education/ai-resources-and-training/ai-image-challenge/RSNA-Pneumonia-Detection-Challenge-2018<br>The gender and age distribution of the examined patients were recorded as 11518 (43.2%) female / 15166 (56.8%) male, and 46.77 +- 16.74 (95%CI) respectively. Lastly, the distribution of radiological findings are presented in Table 4 in the main manuscript.
Recruitment	"NIH compiled the dataset of scans from more than 30,000 patients, including many with advanced lung disease. Patients at the NIH Clinical Center, the nation's largest hospital devoted entirely to clinical research, are partners in research and voluntarily enroll to participate in clinical trials. With patient privacy being paramount, the dataset was rigorously screened to remove all personally identifiable information before release."<br>https://www.nih.gov/news-events/news-releases/nih-clinical-center-provides-one-largest-publicly-available-chest-x-ray-datasets-scientific-community<br><br>The dataset used in this study (N=26,684 - RSNA Pneumonia Challenge training set) is comprised of a subset of this initial data release by NIH, where clinical exams with "no-finding" and "pneumonia-like findings" were randomly selected and re-annotated by radiologists blinded to the initial diagnosis label. https://www.kaggle.com/c/rsna-pneumonia-detection-challenge/discussion/64723
Ethics oversight	The datasets used consist of public and widely used deidentified datasets. The National Institutes of Health (NIH) Clinical Center was responsible for the release of the NIH dataset images. Senior Investigator: Ronald M. Summers, M.D., Ph.D., Senior Investigator of the Clinical Image Processing Service in the Imaging Biomarkers and Computer-Aided Diagnosis Laboratory of the NIH Clinical Center Radiology and Imaging Sciences Department. The authors herein were not involved in the data collection.<br>Additionally, the proposed study was reviewed by the Microsoft Research Ethics Review Program and met all the required ethical considerations outlined by the Office for Human Research Protections, prior to initiation of the research (Reference IRB 10054/ RCT 4375,4374). This was classified as non-human subject research.

Note that full information on the approval of the study protocol must also be provided in the manuscript.
